# Interactome analysis of gene expression profiles identifies CDC6 as a potential therapeutic target modified by miR-215-5p in hepatocellular carcinoma

**DOI:** 10.7150/ijms.51145

**Published:** 2020-10-18

**Authors:** Hongfa Xu, Jianwen Huang, Shengni Hua, Linjun Liang, Xu He, Meixiao Zhan, Ligong Lu, Jing Chu

**Affiliations:** 1 Zhuhai Interventional Medical Center, Zhuhai Precision Medical Center, Zhuhai People's Hospital (Zhuhai hospital affiliated with Jinan University), Zhuhai, Guangdong, 519000, China.; 2 Department of Urology, Zhuhai Interventional Medical Center, Zhuhai Precision Medical Center, Zhuhai People's Hospital (Zhuhai hospital affiliated with Jinan University), Zhuhai, Guangdong, 519000, China.

**Keywords:** bioinformatics analysis, hepatocellular carcinoma, CDC6, miR-215-5p

## Abstract

**Background:** Illustrating the pathogenesis of hepatocellular carcinoma (HCC) pathogenesis as well as identifying specific biomarkers are of great significance.

**Methods:** The original CEL files were obtain from Gene Expression Omnibus, then affymetrix package was used to preprocess the CEL files, the function of DEGs were investigated by multiple bioinformatics approach. Finally, typical HCC cell lines and tissue samples were using to validate the role of CDC6 in vitro. Bioinformatics software was used to predict potential microRNA of CDC6. Luciferase assay was used to verify the interactions between CDC6 and microRNA.

**Results:** A total of 445 DEGs were identified in HCC tissues based on two GEO datasets. GSEA results showed that the significant enriched gene sets were only associated with cell cycle signaling pathway. In the co-expression analysis, there were 370 hub genes from the blue modules were screened. We integrated DEGs, hub genes, TCGA cohort and GSEA analyses to further obtain 10 upregulated genes for validation. These genes were overexpressed in HCC tissues and negatively associated with overall and disease-free survival in HCC patients and related to immune cell infiltration in HCC microenvironments. Finally, Cell Division Cycle 6 (CDC6) was highlighted as one of the most probable genes among the 10 candidates participating in cancer process. The expression of CDC6 either in public datasets and HCC tissues sample were commonly high than the non-cancerous counterpart. Furthermore, we recognized that miR-215-5p, could directly bind to the 3'UTR of CDC6. In addition, CDC6 promoted proliferation via regulation of G1 phase checkpoint and was negative regulated by miR-215-5p to involve in the proliferation of HCC.

**Conclusion:** Our study suggested that CDC6 served as a potential therapeutic target for HCC.

## Introduction

Hepatocellular carcinoma (HCC) ranked the fourth leading cause of mortality in patients suffering from malignant tumors in 2018 1. Mortality from HCC is generally due to metastasis and postsurgical recurrence 2-3. In these tumors, it is well known that there is dysregulation of gene transcription including gene mutations, the activation of oncogenes or the inactivation of tumor suppressor genes that work to promote tumor metastases. Thus, numerous biomarkers involved in HCC genesis have been discovered. However, the identification of key genes associated with HCC have not been completely identified and the molecular mechanisms behind HCC initiation, progression and specific targets for HCC diagnosis and therapy are still needed to be illustrated.

Numerous gene sequencing data have been stored in public databases and widely used with the development of genomics, which provide clues for bioinformatic mining of gene expression profiles involved in cancer. Though there are numerous HCC-associated mRNA microarray data, small sample sizes in individual studies and the diversity of bioinformatic technology platforms have led to substantial discrepancies between research studies. By using integrative analysis of datasets from GEO and TCGA, we successfully produced a cohort of differential expression genes (DEGs) that were focused on the upregulation or downregulation of gene expression between samples that potentially participate in tumorigenesis of HCC.

In this study, the two GEO datasets GSE33006 and GSE84402 were obtained from GEO, containing a total of 16 HCC cases and 16 normal liver cases after quality control. A combined analysis was completed for the two microarray datasets 4-5. The screening of DEGs was analyzed using the limma package 6. The functional pathway analysis including Gene Ontology (GO) term enrichment analysis, and Kyoto Encyclopedia of Genes and Genomes (KEGG) pathway analysis were performed using the cluster profiler package and the Database for Annotation Visualization and Integrated Discovery (DAVID) 7-9. Protein-protein Interaction (PPI) analysis was conducted using the STRING browser and Cytoscape software with cytoHubba app 10-11. Weighted Correlation Network Analysis (WGCNA), a systems biology method for identifying co‑expression networks of DEGs was performed using the WGCNA package 12. GSEA was used to detect whether there were priori defined biological processes enriched in the gene rank derived from DEGs 13. Finally, cell division cycle 6 (CDC6) was observed to be a key candidate gene that may be involved in HCC progression.

Cell Division Cycle 6 (CDC6) is one of ten key candidate genes that play an important role in the assembly of pre-replicative complexes during the G1 phase of the cell cycle, and is a critical determinant of checkpoint mechanisms that coordinate S phase and mitosis 14-15. Repression of CDC6 leads to DNA hyper replication and genomic instability, which results in cell senescence similar to what is caused by oncogene activation 15. The deregulation of CDC6 expression had been reported to be involved in many cancers, such as para-small cell lung carcinomas 16, mantle cell lymphomas 17, prostate cancer 18, Nasopharyngeal carcinoma 19 and breast cancer 20. The full role of CDC6 in HCC remains to be determined.

It had been demonstrated that miRNAs are involved in the occurrence and development of tumors as oncogenes or tumor suppressor genes, and the functions of miRNAs were mediated by binding to the 3'UTR of a target gene, leading to suppression of mRNA translation or degradation of miRNA-bound mRNAs or lncRNAs 21-22. It had been reported that miRNA-215-5p suppresses the progression of papillary thyroid cancer 23 and multiple myeloma 24. To further investigate the role of miRNA-215-5p in the development of HCC, bioinformatics (TargetScanHuman, miRanda database) analysis was used to predict target genes of miRNA-215-5p and uncovered that CDC6 may be a potential target gene of miRNA-215-5p. Despite these findings, evidence for the association between CDC6 and miRNA in HCC remains inconclusive.

Using an integrated analysis of HCC based on DEGs, WGCNA, GSEA as well as the interaction between CDC6 and miRNA-215-5p, the work presented here provides new insights into the mechanisms behind HCC.

## Materials & Methods

### Microarray data collection

The GSE33006 and GSE84402 microarray expression profile of hepatocellular cancer and normal liver tissue, based on the platforms of the GPL570 [HG U133_Plus_2] Affymetrix Human Genome U133 Plus 2.0 Array, were obtained from the GEO (http://www.ncbi.nlm.nih.gov/geo/) database. These two series consists of 17 hepatocellular cancer tissues and 17 adjacent normal liver tissues. Moreover, as for GSE89377 and GSE98383, there contained 40 tumor tissues, 13 para-cancerous tissues, 12 cirrhosis tissues and 11 tumor tissues, 24 para-cancerous tissues, 29 cirrhosis tissues, respectively. Another dataset of hepatocellular cancer profile were obtained from TCGA database containing 374 HCC tumor samples and 50 para-cancerous samples (https://tcga-data.nci.nih.gov/tcga/) and oncomine database to further verify our results.

### Data preprocessing

Raw data for the GSE33006 and GSE84402 datasets were integrated for analysis. Original CEL files were preprocessed by the RMA function in the Affymetrix package of R language [R version 3.3.5] 25. All probe level data in each sample were reduced to a single value using the aggregate function method to determine mean expression values 26. Missing data were assigned by the k-nearest neighbor method 27. The average values of the probes were eliminated as the true level of the gene when numerous probes were mapped to one gene. Quantile normalization and background correction for complete data were performed using the Core package in Bioconductor and the limma R package 28-29. One sample (GSM2233086) was detected as an outlier and removed from subsequent analysis with its adjacent normal tissues.

### Data combining

It is very crucial for combining data from different datasets. The GSE33006 and GSE84402 were chosen to combine for integrated analysis after the raw data were preprocessed due to have the same platform. The combat function in the sva package of R language was use to remove the batch effect of these two datasets 30.

### Screening of DEGs

DEGs between HCC tissues and para-cancerous tissues were identified using the limma package, the statistically DEGs were defined as p<0.01, |logFC| ≥1.6 and the false discovery rate (FDA) < 0.05. Funrich Software (Version 3.1.3, http://funrich.org/index.html) was utilized to detect the DEGs overlapping characteristic among different datasets.

### Functional network establishment of DEGs

To further investigate the function of these DEGs, enrichment of the functions and pathways was analyzed using both online tools of the Database for Annotation Visualization and Integrated Discovery (https://david.ncifcrf.gov/) and cluster profiler, a R package with an analysis and visualization function to extract significant information on the GO and KEGG analyses 31. A p-value <0.05 was set as a significant enrichment. The STRING database (http://string-db.org), an online tool for functional protein-association analyses, was recruited to predict the potential regulatory network to elucidate the key protein in carcinogenesis. Cytoscape software (http://www.cytoscape.org) was employed to construct the graphical network of PPI. The cytoHubba, a plugin of cytoscape used to score and analysis to obtain the optimized parameters to produce the best results for the network. Timer software (https://cistrome.shinyapps.io/ timer/), which includes different types of cancer samples accessible in the TCGA cohort, was used to examine the correlation between expression of the genes and tumor-infiltrating immune cells (TIICs; B cells, CD4+ T cells, CD8+ T cells, neutrophils, macrophages, and dendritic cells). Timer applies a deconvolution method to infer the abundance of TIICs from gene expression profiles.

### Principal component analysis (PCA)

We performed PCA using GEPIA2 websites to distinguish HCC patients from healthy controls based on levels of the key genes. PCA is used to reduce the noisy information and dimensionality of redundant from complex massive datasets. We transformed the original variables into three new orthogonal variables called principal components (PCs). A PC score plot was obtained to represent clear clustering of the target points.

### Gene set enrichment analysis (GSEA)

To identify the potential function of the DEGs, GSEA (http://software.Broadin-stitute.org/gsea/index.jsp) was utilized to analysis whether a series of biological processes which priori defined were enriched in the gene rank between the tumor and para-cancerous groups. The collection of annotated gene sets of c2.cp.kegg.v5.2.symbols.gmt in Molecular Signatures Database (MSigDB) was defined as the reference gene sets.

### Co-expression network

Co-expression network analysis for the DEGs was performed with WGCNA package to construct the correlation of DEGs and to search for the mostly significant correlated gene modules 32-33. The soft thresholding power was set as 14. Gene significance (GS) and module membership (MM) were used to analyze the correlation between module and tumor, the module highly correlated with tumor was selected for further analysis.

### Cell lines and Cell culture

Human HCC cell lines and the normal human hepatic cell line were maintained with 5% CO2 at 37ºC in Dulbecco modified Eagle medium and RPMI 1640 (GIBCO USA), supplemented with 10% fetal bovine serum. All cells were not cultured for more than two months.

### Quantitative real-time PCR

Gene mRNA level were determined by real-time PCR. Total RNA of HCC cells and surgical specimens were extracted by using Trizol reagent (Invitrogen, CA, USA), the reverse-transcribed of mRNA and miRNA using a RevertAid First Strand cDNA Synthesis Kit (Thermo, K1622) and Bulge-LoopTM miRNA qRT-PCR Starter Kit (RiboBio, C10211-1) respectively. β-actin was used as the normalization genes, miR-215-5p expression was normalized to U6. The sequences of PCR primer are listed as follows:

CDC6-forward primer: GGAGATGTTCGCAAAGCACTGG.

CDC6-reverse primer: GGAATCAGAGGCTCAGAAGGTG.

### Immunoblotting analysis

Western blotting was conducted according to the previously described standard methods 34. The primary antibodies included anti-CDC6 (proteintech, 11640-1-AP), Cell Cycle Antibody Sampler Panel (ab228528, abcam), GAPDH (proteintech, 60004-1-Ig).

### Immunohistochemistry analysis

Patient cancer tissues were retrieved from the Chaoxing Biotechnology, China. Immunohistochemical staining of the HCC tissue was carried out following the manufacturer's protocol.

### Prediction and validation of direct interaction between CDC6 and microRNAs

By using online database starBase and TargetScanHuman, we suspected several molecular interactions with CDC6. As analyzed, miR-215-5p showed a potential direct binding site with 3'UTR of CDC6 mRNA. A 500 bp sequence from the 3'UTR of CDC6 mRNA including the putative miR-215-5p binding site was selected as follow:

5'-cacagtaggatcctgcccaataaggagcagcctccccacctcattgtgtttgaggcttggcgccttcctcttaactgtagggcttgagtcaggaacatggcttgactcgcagtggggctgctatgtatcctccctggcttccagccaaaatcacattggtagattcaaaggggccaaatttctttcccctctatctttccctttcccctggttttggaaatagagttttctgtctactgatttgttagtttccttttcttctcccctcactgtcaatttctaggtcattgctgctcttaagactttagcagttggaacagggttggttctgtcaatgatgcatgaagcagacttagtgtccctgcttggcttctgctgcccttgtgggagcaaaagctgatatatgtttgtcagtaaagtcttaagtgtaattcagactgctgaggaagaaagccctttccttgctggcttttctccctgaagctgagagcttcaggaag- 3'.

The corresponding mutant sequence were: 5'-cacagtaggatcctgcccaataaggagc

agcctccccacctcattgtgtttgaggcttggcgccttcctcttaactgtagggcttgagtcaggaacatggcttgactcgcagtggggctgctatgtatcctccctggcttccagccaaaatcacattggtagattcaaaggggccaaatttctttcccctctatctttccctttcccctggttttggaaatagagttttctgtctactgatttgttagtttccttttcttctcccctcagacagttaaagatccagtttgctgctcttaagactttagcagttggaacagggttggttctgtcaatgatgcatgaagcagacttagtgtccctgcttggcttctgctgcccttgtgggagcaaaagctgatatatgtttgtcagtaaagtcttaagtgtaattcagactgctgaggaagaaagccctttccttgctggcttttctccctgaagctgagagcttcaggaag-3'. Sequences above were cloned into pEZX-MT06 luciferase vectors (GeneCopoeia), containing firefly luciferase, and vectors containg renilla luciferase were used for control. SMCC-7721 and Hep3B cells over-expressing miR-215-5p or the negative control were co-transfected with vectors above, and luciferase activity were measured by Dual-Glo Luciferase assay system (Promega, USA) 24h post-transfection.

### Luciferase assay

The duo-luciferase vector pEZX-MT06 (GeneCopoeia) was used to generate luciferase reporter constructs. A 500 bp sequence from the 3-UTR of CDC6 mRNA with wildtype (WT)and mutant type (Mut) putative miRNA binding site were obtain from GeneCopoeia. Cells were seeded in 96-well plates and cotransfected with wild-type or mutated 3'UTR of CDC6 constructs and miR-215-5p mimic for 48h. Luciferase activity was performed by using the Luc-Pair™ Duo-Luciferase HS Assay kit (GeneCopoeia, LF004) according to the manufacturer's protocol.

### Cell cycle assay

Cells were fixed with 70% ethanol at -4°C for 24h. After 24h of fixation and wash with cold 1X PBS twice. Cells were then stained with propidium iodide and RNase A (Beyotime) for 30min at 37°C. Subsequently, cells were analyzed for DNA content using BD FACSCalibur™ flow cytometer (BD Biosciences).

### Cell proliferation assays

Colorimetric MTS assays (Promega; G3580) were performed to determine the viability of HCC cells, as previously reported 34 Briefly, 800 cells/ well were treated in 96-well plate. After various times post-seeding, added 20 μL MTS solution to each well for 3h and the optical density value was calculated at 490 nm.

### Statistical analysis

Statistical analyses were conducted using SPSS (version 17). A Student's test was utilized to assess significance of data from two groups, and one-way analysis of variance (ANOVA) followed by Dunnett's multiple comparison was performed to evaluate differences between multiple groups.The correlations between the CDC6 level and OS and DFS were analyzed with Kaplan Meier survival. All data analysis are represented as the means±S.D., p<0.05 was considered statistically significant difference.

## Results

### Sample preprocessing and DEGs identified in HCC

**Figure 1:**
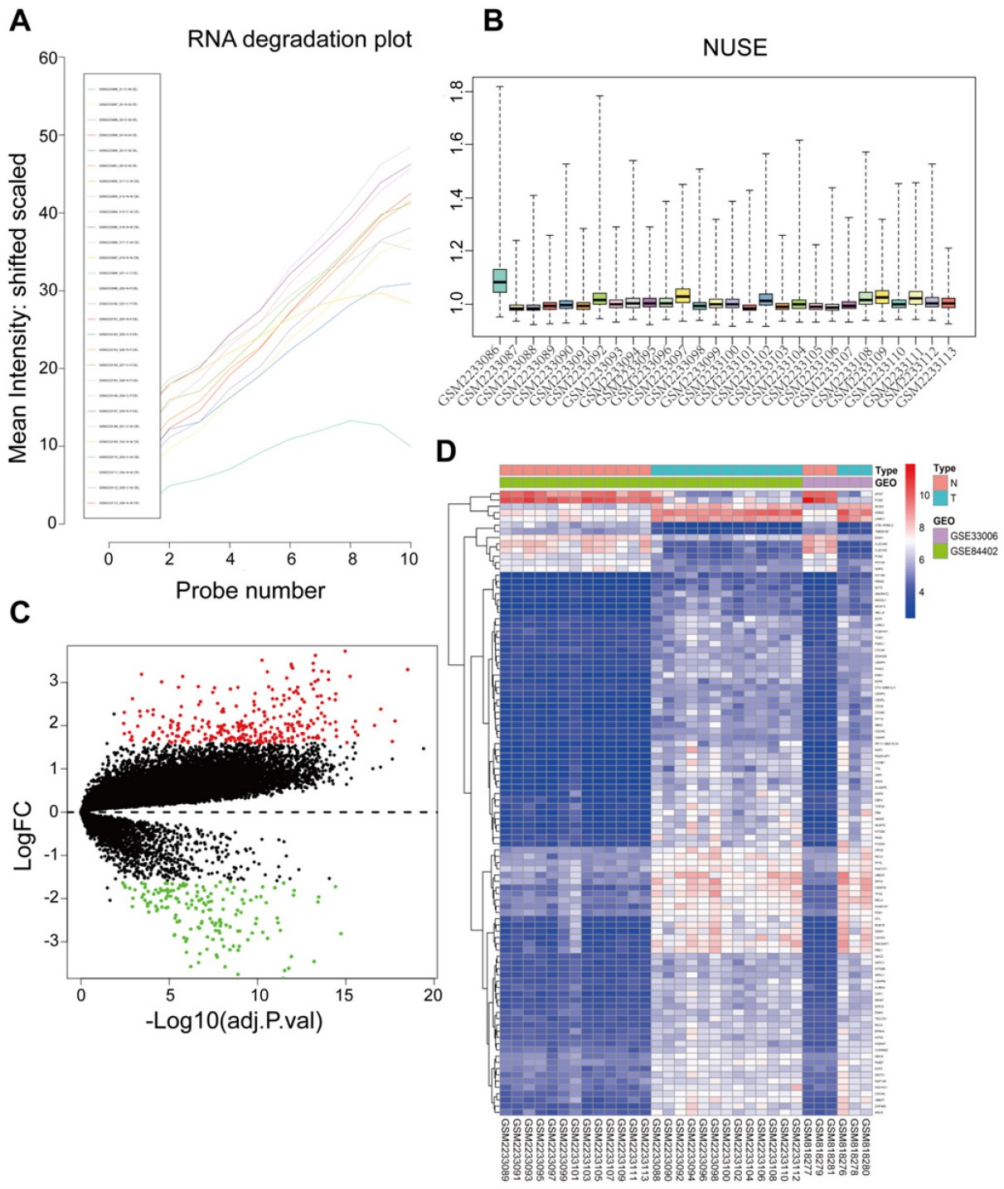
Sample preprocessing and DEGs identified in GSE33006 and GSE84402. (**A**) The RNA degradation of all probe sets were detected using the affy package. The horizontal axis represents fragments of RNA from 5'end to 3'end, while the vertical axis represents the mean fluorescence intensity. GSM2233086 outliers were marked by a red arrow. (**B**) Normalized Unscaled Standard Errors of all probe sets in the datasets were displayed as bar charts through the affy package, the value 1 on the vertical axis means 100% of the untreated control, excellent quality chips have normalized NUSEs and RLEs near or lower than 1 (control value). Poor quality chips have normalized NUSEs and RLEs higher than 1. Only the GSM2233086 NUSE value was obviously higher than controls. Thus GSM22330086 and its corresponding adjacent normal sample GSM2233087 were split. (**C**) A Volcano map of DEGs between HCC tissues and adjacent normal liver tissues. Red dots represented upregulated genes and green dots represented downregulated genes. (**D**) A hierarchical clustering dendrogram of the top 100 DEGs based on the |logFC| value.

Raw data of 34 samples including 16 tumor tissues and 16 adjacent normal tissues were preprocessed and integrated for analysis. One sample (GSM2233086) was detected as an outlier according to the RNA degradation rate and the normalized unscaled standard error values of all probe sets and removed with its adjacent normal tissues from subsequent analysis (Figure 1A and 1B). Thus, there were a total 32 samples used in subsequent analysis. The GSE33006 and GSE84402 datasets were combined by removing batch effects for these two datasets due to having the same platform. Based on the Limma R package, microarray data for a total of 21654 genes from 32 samples were obtained after data preprocessing. Based on cut‑off criteria, a total of 445 DEGs were identified, including 284 upregulated genes and 161 downregulated genes in HCC compared to normal liver tissues (Figure 1C). The top 100 significant DEGs were also visualized on a heatmap based on the level of |logFC| (Figure 1D).

### Functional enrichment analysis and PPI network construction

**Figure 2:**
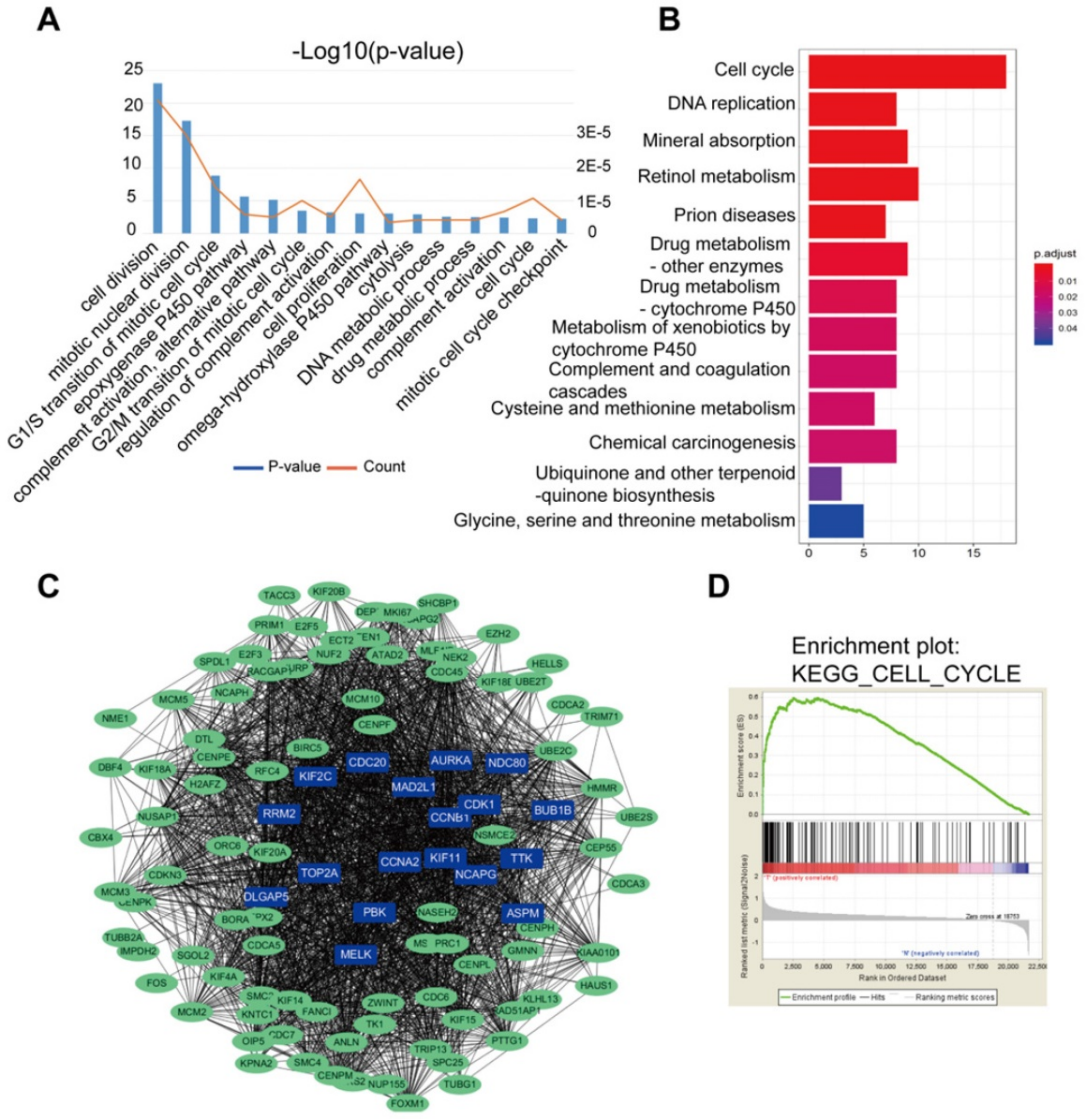
DEGs of the two datasets based on GO, KEGG pathway, PPI nework and GSEA. (**A**) The top 15 GO annotations included biological process in the enrichment analysis of the DEGs. (**B**) The significant KEGG pathways in the enrichment analysis of DEGs. (**C**) Some DEGs were filtered into the PPI network that contained 108 nodes and 2369 edges. The color intensity in each node was proportional to change fold of expression compared to para-cancerous samples. (**D**) Gene set enrichment analysis (GSEA) for HCC samples and adjacent normal tissues. GSEA showed that significantly enriched gene sets were associated with cell cycle, p<0.05.

Results of the GO analysis represented that cell division, mitotic nuclear division, G1/S transition of mitotic cell cycle were the top 3 significant biological processes assigned to DGEs (Figure 2A). In the KEGG analysis, DEGs gathered in the cell cycle, DNA replication and mineral absorption (Figure 2B). In addition, part of the DEGs were filtered into the PPI network complex to analyze the interactive relationship based on STRING databases, where statistical significance made up 108 nodes and 2369 edges (Figure 2C). Next, DGEs were analyzed by GSEA. GSEA was performed to search for KEGG pathways enriched in HCC samples compared to adjacent normal samples. GSEA results showed that significant enriched gene sets were only associated with cell cycle signaling pathways (p<0.05) (Figure 2D), where there were a total of 66 genes enriched in the signaling pathway.

### Gene set enrichment and co-expression analyses

**Figure 3:**
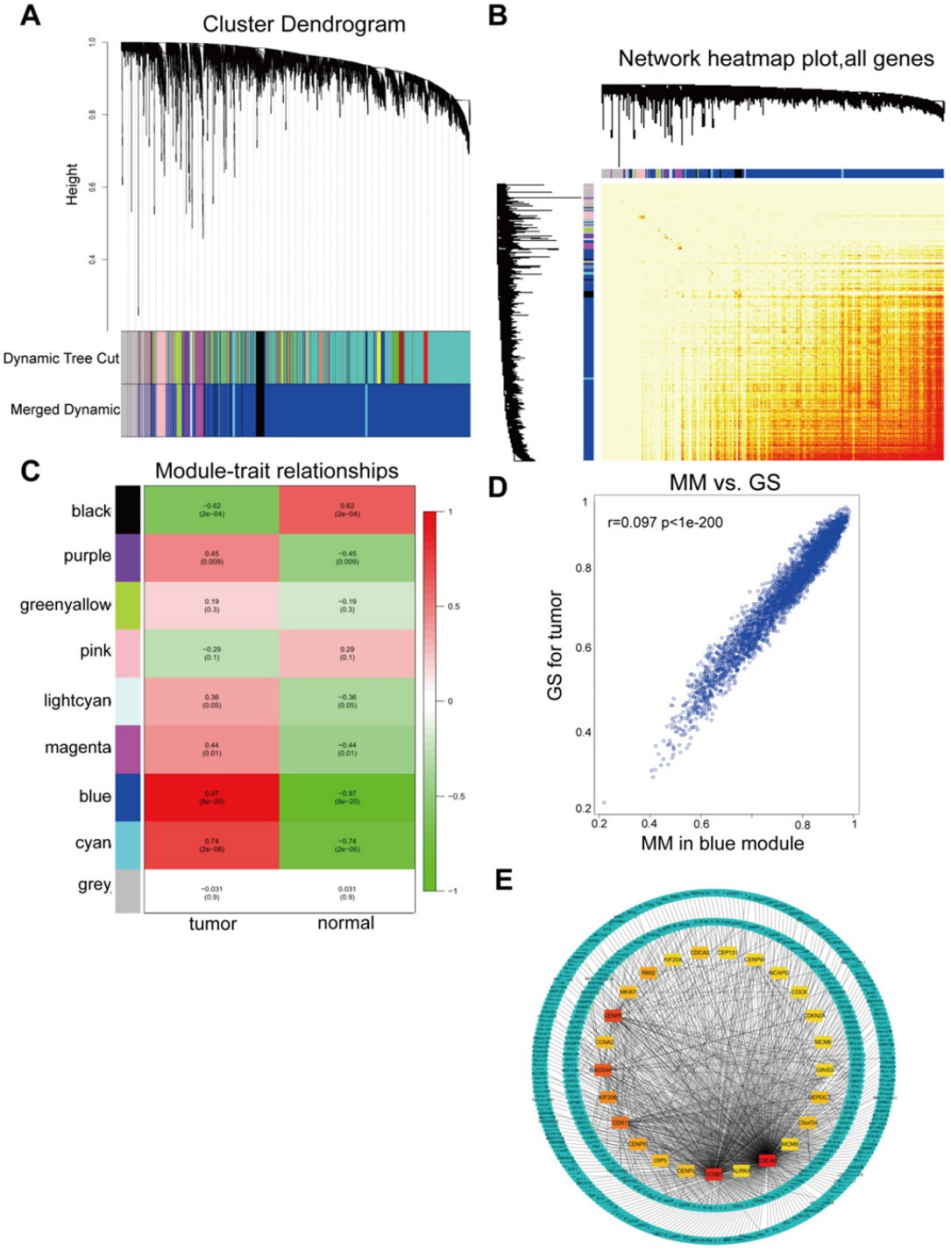
WGCNA of GSE29570 and GSE89657 datasets. (**A**) Differentially expressed genes in 32 samples of HCC and adjacent para-cancerous were assigned to one of 9 moduleswith a cutoff powers =14. The top image represented all gene dendrograms, the bottom image represented gene modules with different colors. (**B**) Correlation between modules eigengenes and sample features. Each cell contained the corresponding correlation coefficient and P value. Among these, the blue and black modules were the most probable relevant modules with cancer traits. (**C**) A scatter diagram of the correlation between GS for HCC and the MM in the blue module. Intra-modular analysis of the genes in the blue module that show a strong relationship to HCC, with p<1e-200 and r =0.97. (**D**) A heatmap of all DEGs. The intensity of the red color represented the strength of the relationship between pairs of modules on a linear scale. (**E**) PPI network of hub genes identified by co-expression network analysis in the blue module. The color intensity of each node was proportional to change fold of expression in comparison to para-cancerous samples. The network contained 370 nodes and 3933 edges.

All DGEs were analyzed by WGCNA. WGCNA was used to detect modules of highly correlated genes. Genes in each group with highly similar expression patterns were combined into modules using average linkage hierarchical clustering. A power of 20 was selected as the soft-thresholding to ensure a scale-free network. A total of 9 modules were excavated (Figure 3A) and a corresponding heatmap of all genes was shown (Figure 3B). The association between each module and tumor traits were demonstrated in each group, as shown Figure 3C. The blue module was the most relevant module with tumor traits (p=8x10^-20^, R^2^=0.97), which was extracted for further analysis. Furthermore, a scatter diagram of the correlation between GS for HCC and the MM in the blue module were shown (Figure 3D). GS and MM of genes in the blue module showed a correlation with HCC. Hub were functionally significant in biological processes 35. Genes in a module were selected for candidate hub genes with a cutoff of weighted correlation coefficient s≥0.8. Therefore, 370 genes with high connectivity in the blue module were further analyzed. Furthermore, the PPI network for hub genes in the blue module was generated according to the STRING database and the top 25 hub genes in the co-expression network were screened (Figure 3E).

### Functional analyses of key genes

**Figure 4:**
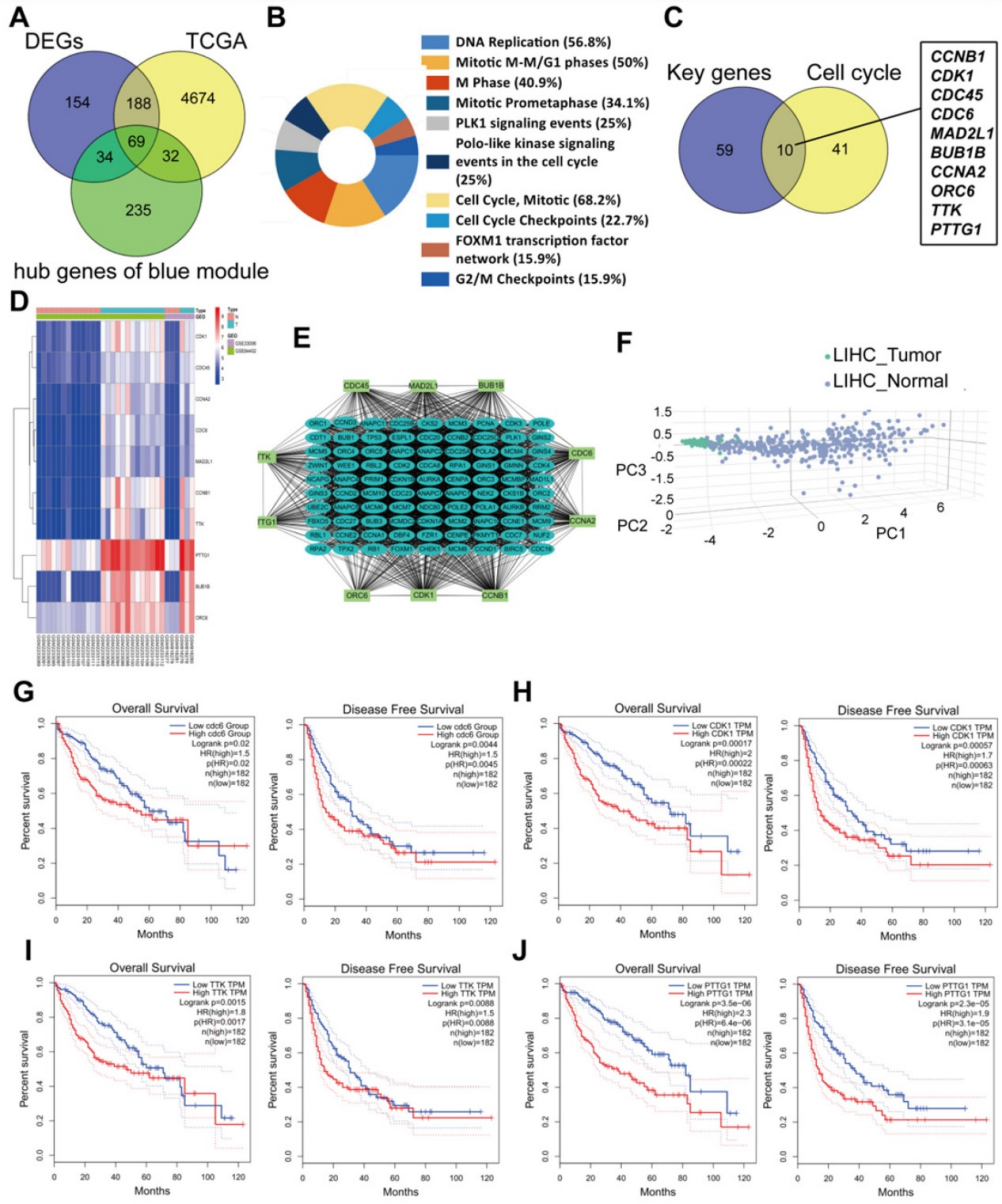

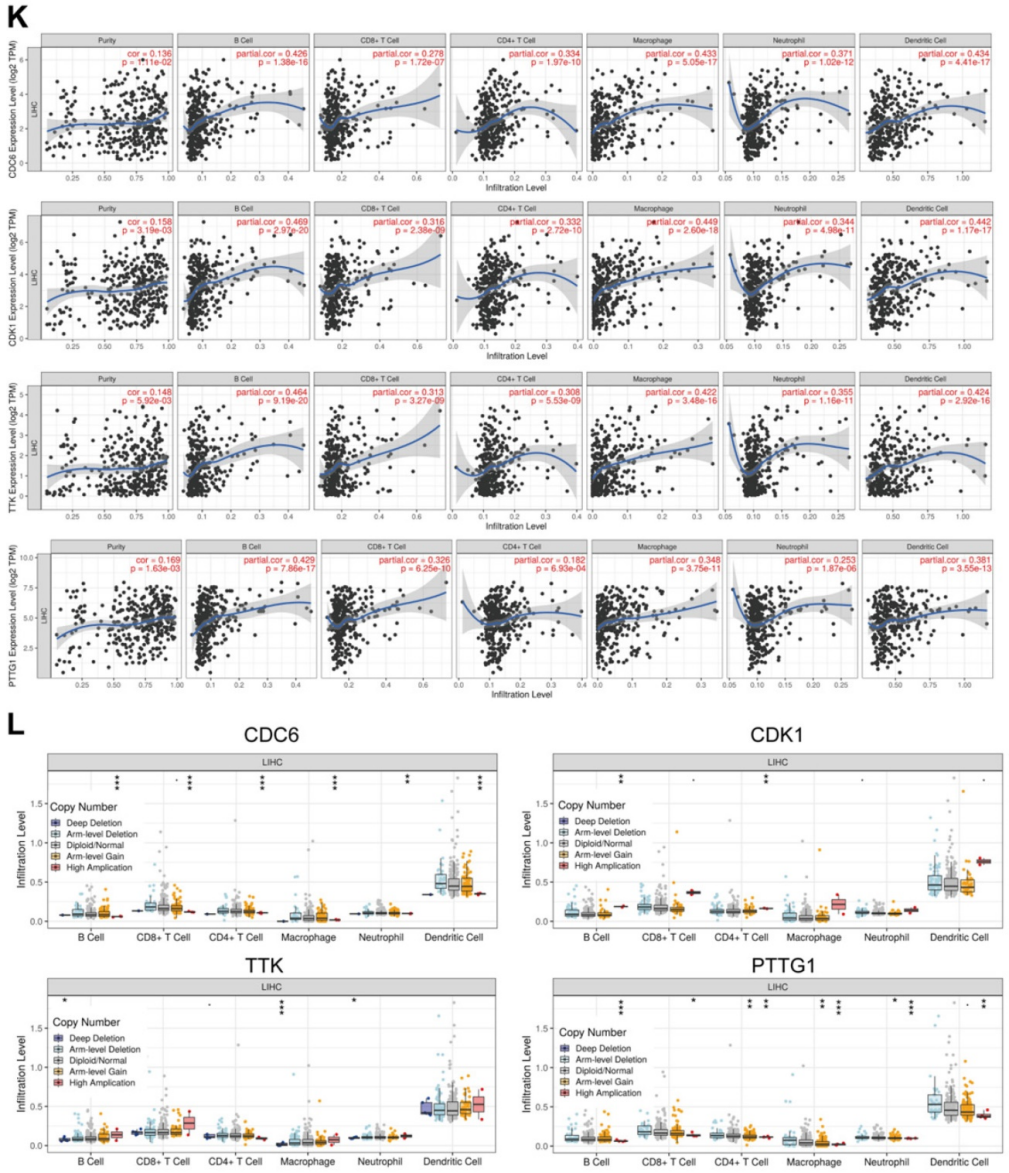
Molecular functional pathways and processes of 10 key genes. (**A**) A Venn diagram of DEGs from GEO, DEGs from TCGA and hub genes in the Brown module revealed 69 key genes. (**B**) Significant KEGG pathways in the enrichment analysis of 69 key genes. (**C**) A Venn diagram of 69 key genes and genes involve in the cell cycle pathway using GSEA revealed 10 genes involved in cell cycle modulation. (**D**) A hierarchical clustering dendrogram of 10 genes based on the value of |logFC|. (**E**) A PPI network of 10 genes. Many genes involved in the progression of HCC interact with 10 key genes. (**F**) A three-dimensional principle component analysis (PCA) score plot showing that HCC patients can be effectively distinguished from healthy controls based on the expression of these 10 genes. (**G-J**) Analysis of the correlation between CDC6 (**G**), CDK1 (**H**), TTK (**I**) and PTTG1 (**J**) gene expression signatures and overall survival (OS) and disease-free survival (DFS) for HCC patients included in the TCGA cohort. (**K**) Correlation between CDC6, CDK1, TTK and PTTG1 levels and tumor purity, infiltrating levels of CD8+ T cells, CD4+ T cells, macrophages, neutrophils and dendritic cells in hepatocellular carcinoma tissues.Each black dot represents one sample in the TCGA cohort. (**L**) CDC6, CDK1, TTK and PTTG1 copy number variation affected infiltrating levels in hepatocellular carcinoma tissues.

Common differently expressed genes from the DEGs of GEO and TCGA and hub genes in the Brown module were overlapped. There were 69 differently expressed genes screened (Figure 4A). KEGG pathway analyses of the 69 genes was performed by DAVID. Significantly enriched signaling pathways were in DNA replication, mitotic M-M/G1 phases, cell cycle mitosis, cell cycle checkpoint and G2/M damage checkpoint (Figure 4B). This demonstrated that cell cycle regulation plays the most important role in HCC progression. Thus, we further overlapped the 69 genes with genes involved in the cell cycle pathway using GSEA and found that there were 10 genes CCNB1/CDK1/CDC45/CDC6/ORC6/MAD2L1/BUB1B/CCNA2/TTK/PTTG1 screened (Figure 4C). These 10 genes were regarded as “real” key genes for HCC progression. Analysis of the expression fold-change of the 10 key genes showed strong significant differences at mRNA level between tumor and normal liver tissues, which was also visualized on a heatmap in Figure 4D. In addition, an initial unstable plaque PPI network was constructed using 10 key genes as input for STRING databases and Cytoscape software. The 10 key genes showed distinguished networks and interactions (Figure 4E). Furthermore, based on the expression of these 10 genes, we could effectively distinguish HCC patients from healthy controls in the TCGA cohort using PCA analysis (Figure 4F). We then further determined the association of these 10 key genes with HCC patient prognosis. Levels of these 10 genes were negatively correlated with OS and DFS for HCC patients from the TCGA cohort (Figure 4G-4J, Supplementary Figure 1A-1F). We also performed an interrelation analysis comparing infiltrating immune cells in HCC tissues and the expression of these 10 genes. The expression levels of these 10 genes were positively related to the infiltration levels of tumor purity, B cells, CD8+ T cells, CD4+ T cells macrophages, neutrophils and dendritic cells in HCC tissues. This showed that higher expression levels of these 10 genes suggested an advantage for cancer immunotherapy (Figure 4K, Supplementary Figure 2A-2F). In addition, these genes had copy number variations significantly correlating with infiltrating levels of B cells, CD4+ T cells, macrophages, neutrophils and dendritic cells (Figure 4L, Supplementary Figure 3A-3E), except for ORC6 due to not included in the database.

### CDC6 served as a biomarker and prognostic factor for HCC

**Figure 5:**
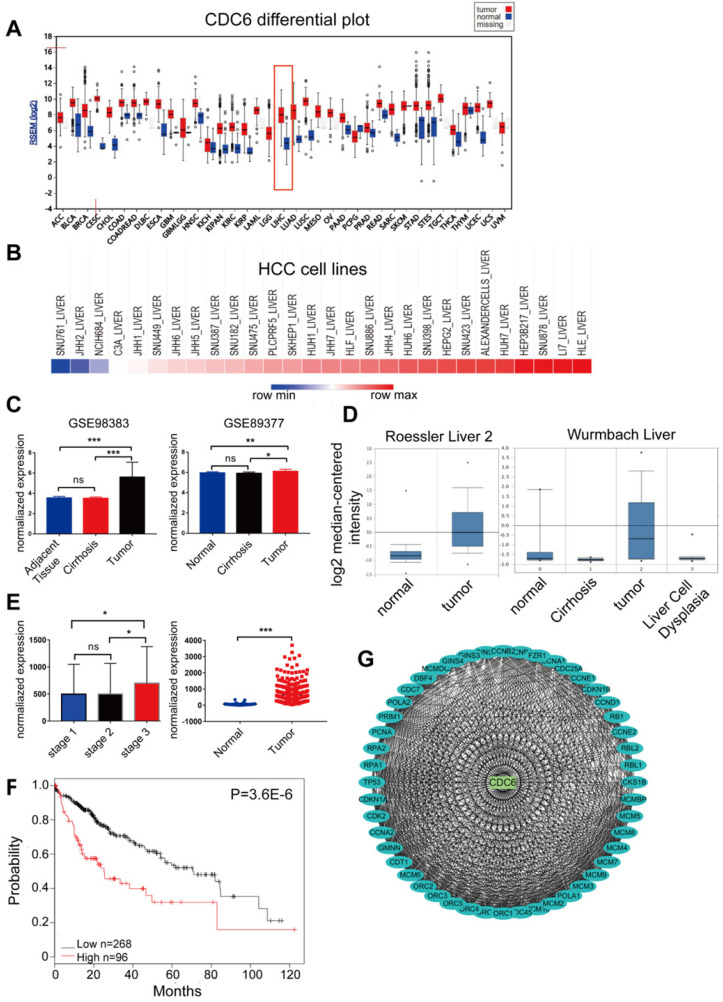
CDC6 expression profiles in GEO/TCGA/Oncomine datasets in human malignancies. CDC6 served as a biomarker and prognostic factor for HCC. (**A**) Most human malignancies expressed relatively higher CDC6 levels compared to para-cancerous tissues in a reliable confidence interval from the Firebrowse database (http://firebrowse.org/). (**B**) CDC6 expression in HCC cell lines from the CCLE database (https://portals.broadinstitute.org/ccle/). (**C-E**) Validation of CDC6 mRNA expression from both GEO databases (**C**), Oncomine (**D**) and TCGA (**E**). (**F**) OS of HCC cohorts based on the Kaplan-Meier plotter database. (**G**) A PPI network of CDC6 identified by co-expression network analysis.

CDC6, one of the 10 candidate key genes, plays an important role in the progression of some cancers 16 - 20, but the full role of CDC6 in HCC still remains unclear. We then searched the CCLE and Firebrowse online database to exam the expression of CDC6. A comprehensive profile of CDC6 was amplified in many human malignancies (Figure 5A). We further detected the expression variation of CDC6 between HCC cell lines. As demonstrated, the expression of CDC6 in differential HCC cell lines was high (Figure 5B). Then, GEO/TCGA/Oncomine datasets were utilized to further validate the expression of CDC6. Results showed that CDC6 expression was higher in HCC tissues compared to para-cancerous tissues (Figure 5C-5E). Based on the Kaplan-Meier Plotter online database, OS was longer for patients with low CDC6 expression compared to those with high CDC6 expression (Figure 5F). Furthermore, the PPI network based on CDC6 was analyzed. As expected, CDC6 represented a complex protein protein interaction as shown in Figure 5G, Many proteins involved in the progression of HCC interaction with CDC6, including TP53, RB1 and MCMs family members. Together, these analyses may reveal that CDC6 was associated with extent of malignancy and clinical stage of OC and may serve as a potential therapeutic target for HCC.

### CDC6 is expressed at aberrantly high levels in HCC tissues and cell lines and serves as a miR-215-5p direct target gene

**Figure 6:**
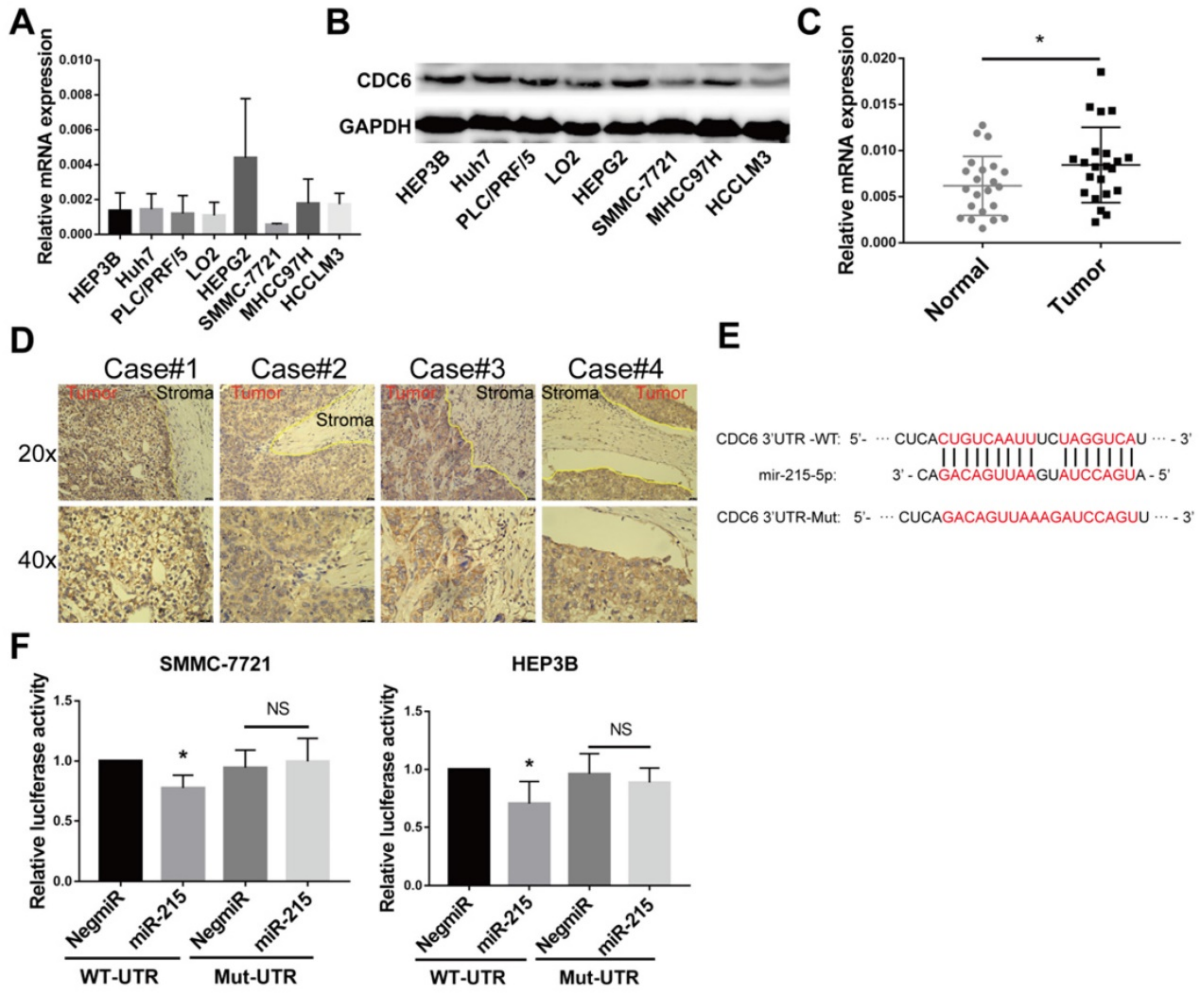
CDC6 expression was further verified in HCC patient specimens and cell lines and serve as a direct target gene for miR‑215-5p. (**A-B**) The relative CDC6 mRNA and protein levels were determined using qPCR(A) and immunoblotting (**B**) in HCC cell lines. (**C**) The mRNA levels of CDC6 (normalized to β-actin) in HCC tissues and para-cancerous tissues were confirmed using quantitative real-time PCR. (**D**) CDC6 protein levels in HCC tissues were analyzed by IHC, using yellow lines to indicate the tumor and stroma edges. (**E**) Scheme for the predicted miR-215-5p binding site in the wild type CDC6 mRNA 3'UTR (3'UTR-WT) and in the mutant construct (3'UTR-MUT). (**F**) Relative luciferase activities in HCC cells co-transfected with pEZX-CDC6-WT and miR-215-5p vs. control vector. Firefly luciferase activity was normalized to Renilla luciferase. Student's t-test, Data represent mean ± S.D (from triplicates); *p<0.05.

The expression of CDC6 was assessed in HCC using RT-qPCR and immunoblottings. Up-regulation of CDC6 was observed in multiple human HCC cell lines compared to immortalized normal liver epithelial cells LO2 (Figure 6A and 6B). Meanwhile, CDC6 mRNA expression levels were also detectable by RT-qPCR in HCC tissue samples and para-cancerous tissues. The expression levels of CDC6 were relatively higher in HCC tissues compared to para-cancerous tissues (Figure 6C). We next performed immunohistochemistry staining for CDC6 in liver tumors and revealed that CDC6 was highly expressed in tumors compared to stroma tissues. This demonstrated dysregulated expression and a pro-invasion role for CDC6 in liver cancer (Figure 6D).

Bioinformatics analysis using miRanda and TargetScan databases revealed that CDC6 was a target gene of miR-215-5p (Figure 6E). To confirm CDC6 was a target gene of miR-215-5p, the 3'UTR sequence of the wide type CDC6 gene and the corresponding control luciferase vectors containing a mut-type CDC6 gene was inserted into a dual luciferase reporter vector pEZX-MT06. Then, pEZX-MT06 and miR-215-5p mimics or a miR-negative control were transfected into HCC cells. As shown in Figure 6F and 6G, after transfection with a miR-215-5p mimic and pEZX-CDC6-wt, the luciferase activity was significantly inhibited compared to the miR-negative control and pEZX-CDC6-wt groups. Transfection with a miR-215-5p mimic and pEZX-CDC6-mut, the suppressive effect induced by miR-215-5p, was significantly abolished. These findings suggested that miR-215-5p serviced as a regulator direct binding to the 3'UTR of CDC6 mRNA to negatively regulate CDC6 expression.

### CDC6 promoted proliferation via regulation of G1 phase checkpoint and was negative regulated by miR-215-5p to involve in the proliferation of HCC

**Figure 7:**
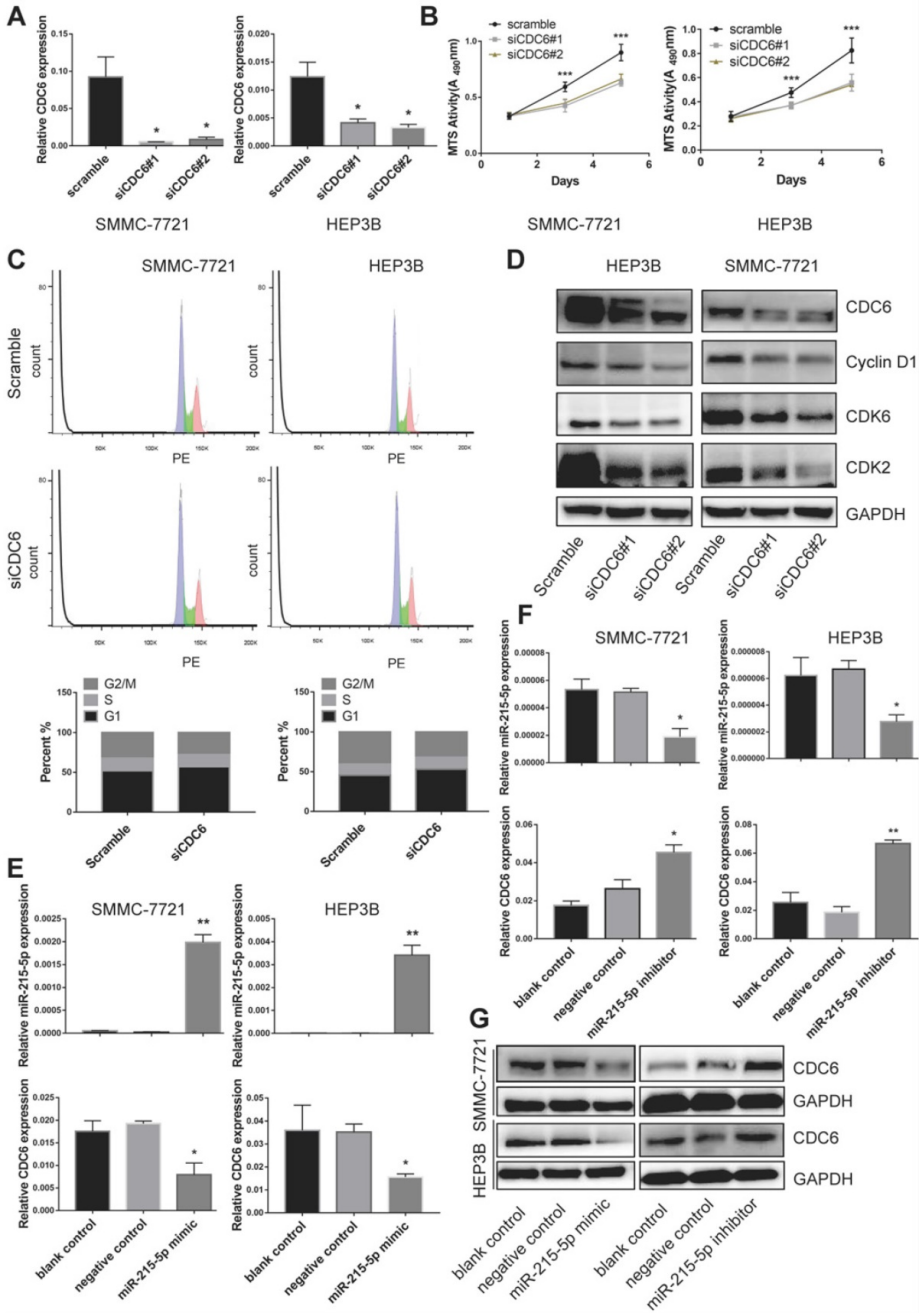
miR-215-5p service as a regulator to negative regulate CDC6 expression. (**A**) Relative level of CDC6 in HCC cell were verified after knockdown of CDC6. (**B-C**) viability (**B**) and Cell cycle distribution (**C**) of HCC cell lines were verified after knockdown of CDC6. (**D**) The protein levels of cell cycle-related proteins were verified by western blot assay. (**E-G**) Relative level of miR-215-5p and CDC6 in HCC cells were verified after transfection with miR-215-5p mimic or miR-215-5p inhibitor. *p<0.05, **p<0.01, and ***p<0.001.

As that CDC6 was highly expressed in HCC tissue as well as cell lines, we then further wondered whether CDC6 was involved in the proliferation of HCC cells in vitro. We generated transiently transfected SMMC-7721 and HEP3B cell lines with scramble control and siRNA, and the CDC6 expression level was confirmed using real-time PCR (Figure 7A). We observed that compared with the scramble control group, the CDC6 suppression resulted in inhibited the proliferation of both cell lines growth (Figure 7B). To further explore the mechanism of CDC6 on HCC proliferation, cell cycle distribution of both cell lines was analyzed by flow cytometry. Results showed that knockdown of CDC6 contributed to G1 phase arrest (Figure 7C). Furthermore, we then explored whether CDC6 suppression could influence the expression of cell cycle-related proteins including cyclin D1, CDK6 and CDK2. As showed in figure 7D, western blot assay demonstrated CDC6 could regulate the expression of cell cycle-related proteins. From these data, we could conclude that CDC6 suppression had an inhibitory effect on HCC cell proliferation via modulation of G1 phase checkpoint.

To explore the relationship between CDC6 and miR-215-5p, we then transfected both cell lines with miR-215-5p mimic or miR-215-5p inhibitor. The miR-215-5p expression levels were confirmed using RT-qPCR (Figure 7E and 7F). The mRNA and protein level of CDC6 were determined by RT-PCR and western blotting, respectively. Results showed that the mRNA level and protein expression of CDC6 was obviously downregulation after transfected with miR-215-5p mimic (Figure 7E and 7G), meanwhile, opposing effects could be found after transfected with miR-215-5p inhibitor (Figure 7F and 7G). Thus, these data clearly indicated that CDC6 was negative regulated by miR-215-5p in HCC.

## Discussion

HCC is a highly aggressive neoplasm. It is common that patients suffering from HCC are diagnosed at a relatively later stage. Issues with radical resection, ionizing radiation resistance and chemotherapy resistance poses a challenge in the HCC therapeutic strategy 36, 37. Currently, the therapeutic strategy focused on molecule-targeting has shown remarkable benefits for patients 38, 39. However, there are few effective molecule-targeted drugs approved in clinical practice 38. Furthermore, the molecular mechanisms where cancer cells develop multi-drug resistance and biomarkers of HCC have not been fully elucidated. It is difficult for clinicians to judge the condition of the patient or predict patient prognosis. It is urgent to identify reliable biomarkers, which are meaningful in preclinical and clinical practice and to develop guidelines for precise and personal cancer therapy.

Numerous molecules had been aberrantly expressed in HCC. These molecules connect and establish complex networks involving HCC genesis and progress. Here, by data mining from NCBI GEO and TCGA databases, we obtained two series from GEO datasets and performed an integrated analysis to try to identify valuable clues. There was a total of 16 HCC cases and 16 normal liver cases data available after quality control measures and combined analysis of the two microarray datasets. A total of 445 DEGs were identified, including 284 significant over-expression genes and 161 downregulated genes in HCC compared to normal liver tissues. GO and KEGG enrichment analyses were subsequently conducted to further analyze the function of these genes. Combined with the results of enrichment, a set of pathways involved in tumorigenesis, were identified, including cell cycle, cell proliferation and chemical carcinogenesis. A total of 21654 genes were analyzed by GSEA and WGCNA. The cell cycle signaling pathway was enriched by GSEA. According to WGCNA analysis, 21654 genes were clustered into 9 modules. Blue and black modules were the most relevant modules to tumor traits, especially genes in blue modules. Genes in the blue module were selected as hub genes with a cutoff of correlation 0.8 and a total of 370 hub genes were identified.

Furthermore, the DEGs were overlapped between the GEO, TCGA. significantly enriched genes involved in the cell cycle pathway and hub genes in the blue module. A map of 10 key genes overlapping in all datasets was obtained. Thus, a comprehensive view of the molecular function and pathway of these 10 candidates was obtained. Our data suggested that higher expression levels of these 10 genes indicate an advantage for checkpoint inhibitor therapy, potentially providing guidance for cancer immunotherapy. To better screen prognostic and diagnostic markers, we also identified these genes for where expression precisely predicted patient prognosis and suggested their relevance to HCC pathogenesis and progression.

CDC6, an essential regulator of DNA replication and one of 10 candidates, was noticed during our analyses. CDC6 is located at chromosome 17q21.3 and the expression of CDC6 is cell cycle regulated. It is expressed in late mitosis once cells are rapidly replicating and expression is regulated by Mcm1 15, 40. CDC6 has been shown to determine the rate of initiation of DNA replication through interaction with ORC6p 41. E2F may regulate the expression of CDC6 and Orc1, required for the induction of cellular DNA synthesis 42. Considering that CDC6 played an important role in DNA replication and cell cycle regulation, its deregulation may have a negative impact on genomic integrity and induce malignant proliferation of cells. High expression of CDC6 protein was reported in most cancers. There have also been reports that CDC6 was downregulated in aggressive prostate cancer 44 and some cases of non-small cell lung carcinomas 45. This accumulating evidence validated that CDC6 has differential expression profiles and functions in various malignancies. However, the exact role of CDC6 involved in HCC pathogenesis and its underlying molecular mechanisms remain poorly understood.

In this study, we first confirmed the CDC6 over-expression profile in HCC tissues by integrating bioinformatic analyses. In addition, CDC6 was highly expressed in most HCC cell lines and tumors as shown using the Firebrowse and CCLE database. Elevated expression of CDC6 in HCC tissues was associated with advanced T stage as shown by the TCGA database. Kaplan—Meier analysis revealed that high expression of CDC6 served as a poor prognosis factor for HCC. Moreover, the relationships between CDC6 expression and tumor-infiltrating immune cells are still unknown. For the roles of immune and inflammatory response, the correlation between CDC6 expression and the marker sets of immune cells indicated CDC6 played an important role in regulating tumor immunology. Our results suggested CDC6 expression had strong correlations with tumor purity, infiltrating levels of CD8+ T cells, CD4+ T cells, macrophages, neutrophils and dendritic cells in hepatocellular carcinoma tissues. But how CDC6 influenced tumor-infiltrating immune cells in hepatocellular carcinoma should be further explored. The strong correlations between CDC6 and immune cells in HCC suggested CDC6 may play a major role in HCC progression. Then to further validate our findings via public database mining, we conducted research using HCC patients specimens and tumor cell lines. As expected, CDC6 was over expressed in HCC tissues and tumor cell lines compared to counterparts using RT-qPCR and IHC assays. This suggested highly reliability of database mining analyses.

Bioinformatics analysis revealed CDC6 as a potential target of miR-215-5p using the targetScanHuman and miRanda databases. It had been reported that miR-215-5p was involved in various pathological processes including proliferation, mesenchymal transition, metastasis and apoptosis 22-24. However, relative research regarding miR-215-5p and CDC6 in HCC was not sufficient. Due to a potential binding site of CDC6 mRNA 3'UTR, which could interact with miR-215-5p, we hypothesized a direct interaction between the two molecules. Wildtype and mutated 3'-UTR sequences of CDC6 were constructed and luciferase signals were measured using a dual-luciferase reporter assay. As expected, miR-215-5p directly bound to the CDC6 3'UTR to negatively modulate CDC6 expression. Then to explore the function of miR-215-5p and CDC6 and their relationship in HCC, we knocked down CDC6 or transfected with miR-215-5p mimic or inhibitor and then the levels of CDC6 and miR-215-5p were determined. Knockdown of CDC6 reduced the cell vitality of HCC cells and caused the cell cycle of HCC cells arrest in G1. miR-215-5p inhibitor significantly decreased the level of miR-215-5p and increased the expression of CDC6, opposing effects could be found in transfecting with miR-215-5p mimic cells. Nevertheless, the full function and molecular mechanisms behind CDC6 in HCC still require additional investigation.

## Conclusion

According to the discovery of our study, we had determined that CDC6 had an important role in the tumorigenesis of HCC and provided a new insight into the mechanism of HCC. Thus CDC6 could serve as a useful biomarker for improving the prediction of HCC patient prognosis.

## Supplementary Material

Supplementary figures.
