# Supplementary material for: Interactome analysis of gene expression profiles identifies CDC6 as a potential therapeutic target modified by miR-215-5p in hepatocellular carcinoma
Source: Int J Med Sci. 2020 Oct 18;17(18):2926–40. doi: 10.7150/ijms.51145 (PMC7646103; doi:10.7150/ijms.51145)
Supplement: Supplementary file 1 — Supplementary figures. [file ijmsv17p2926s1.pdf]

SUPPLEMENTARY MATERIALS

Supplementary Figures

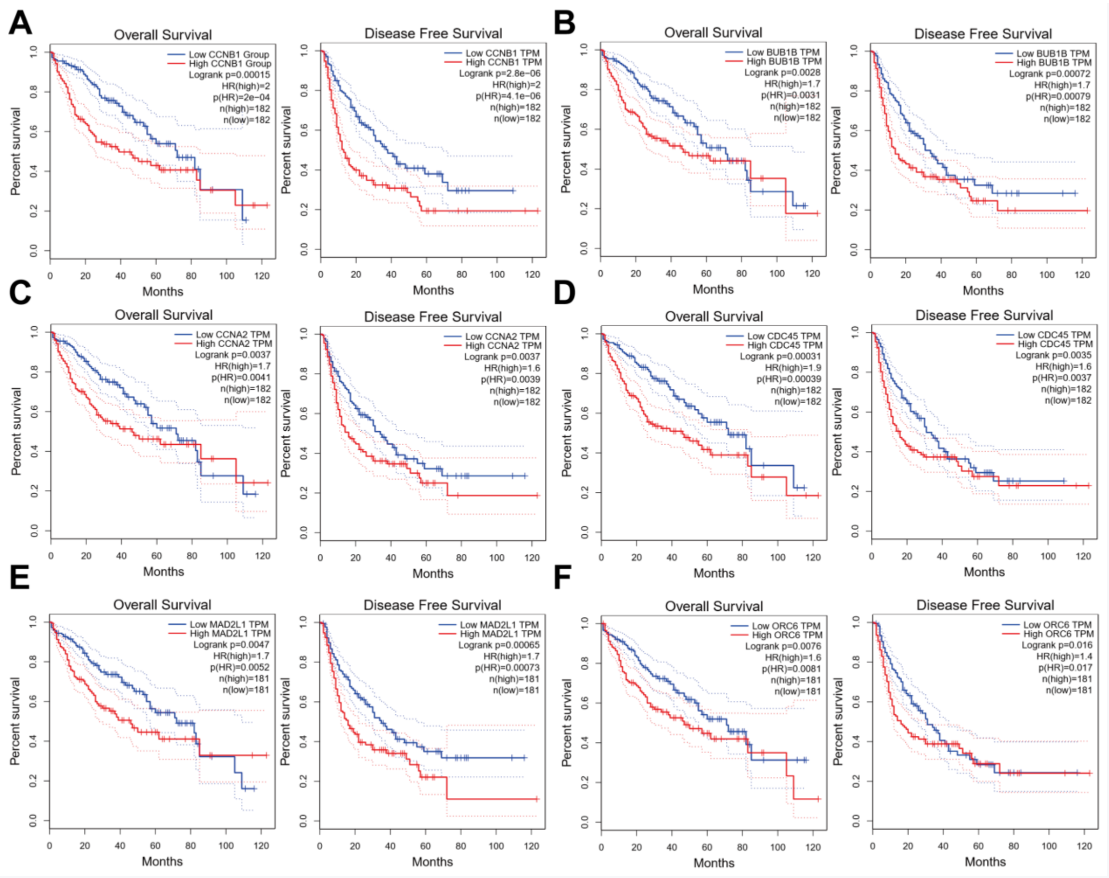

Supplementary Figure 1. Survival analysis based on key genes. (**A–F**) Survival analysis of the association between CCNB1 (**A**), BUB1B (**B**), CCNA2 (**C**), CDC45 (**D**), MAD2L1 (**E**) and ORC6 (**F**) expression and overall survival or disease-free survival based on hepatocellular carcinoma patients in the TCGA cohort.

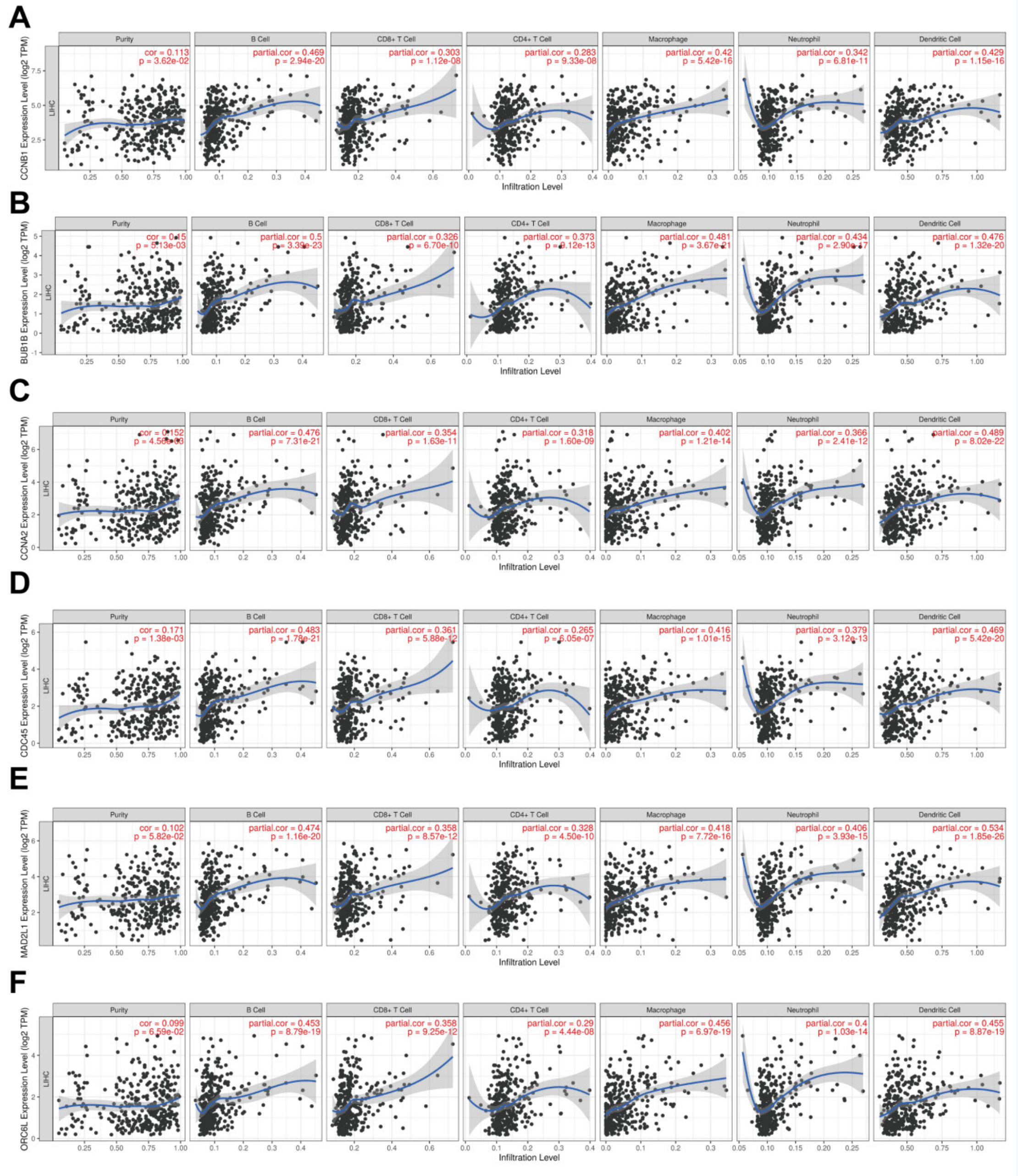

Supplementary Figure 2. (**A–F**) Correlation between levels of CCNB1 (**A**), BUB1B (**B**), CCNA2 (**C**), CDC45 (**D**), MAD2L1 (**E**) and ORC6 (**F**) and tumor purity, infiltrating levels of CD8+ T cells, CD4+ T cells, macrophages, neutrophils and dendritic cells in hepatocellular carcinoma tissues. Each dot represents a sample in the TCGA cohort.

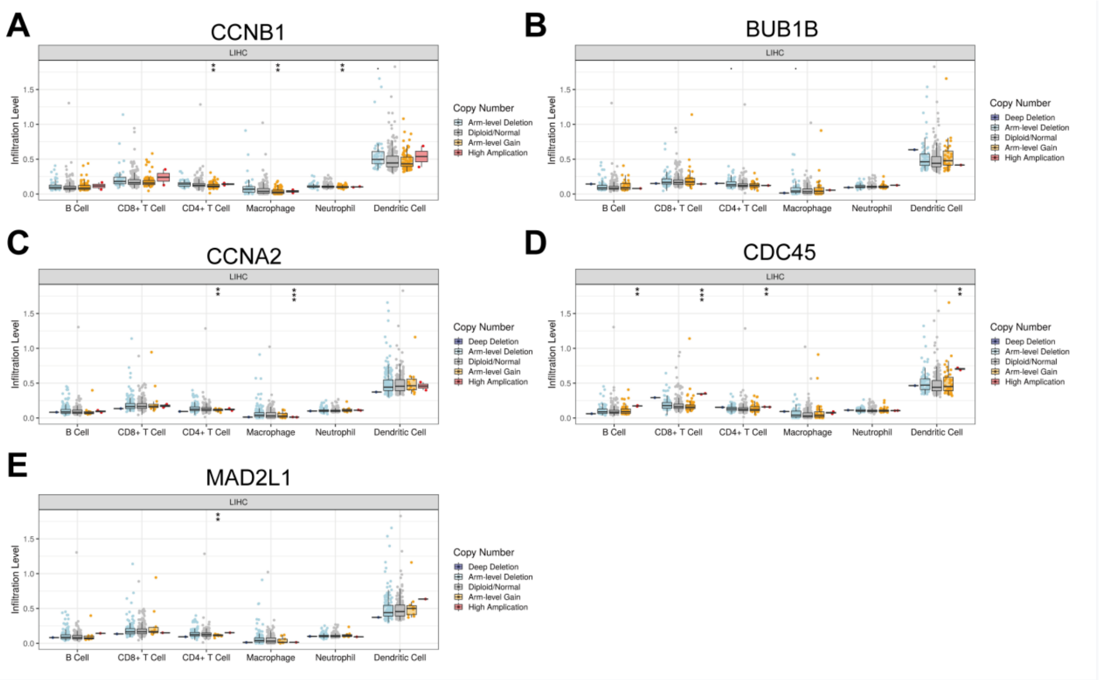

Supplementary Figure 3. (**A–E**) Correlation between copy number variation of key genes and immune cell infiltration. Correlation between CCNB1 (**A**), BUB1B (**B**), CCNA2 (**C**), CDC45 (**D**) and MAD2L1 (**E**) copy number variation affected infiltrating levels in hepatocellular carcinoma tissues.
